# Performance of Multilevel Methods for Excited States

**DOI:** 10.1021/acs.jpca.2c05013

**Published:** 2022-09-12

**Authors:** Bence Hégely, Ádám B. Szirmai, Dávid Mester, Attila Tajti, Péter G. Szalay, Mihály Kállay

**Affiliations:** † Department of Physical Chemistry and Materials Science, Faculty of Chemical Technology and Biotechnology, Budapest University of Technology and Economics, Műegyetem rkp. 3, H-1111 Budapest, Hungary; ‡ Laboratory of Theoretical Chemistry, Institute of Chemistry, ELTE Eötvös Loránd University, P.O. Box 32, H-1518 Budapest 112, Hungary; ¶ ELKH-BME Quantum Chemistry Research Group, Műegyetem rkp. 3, H-1111 Budapest, Hungary

## Abstract

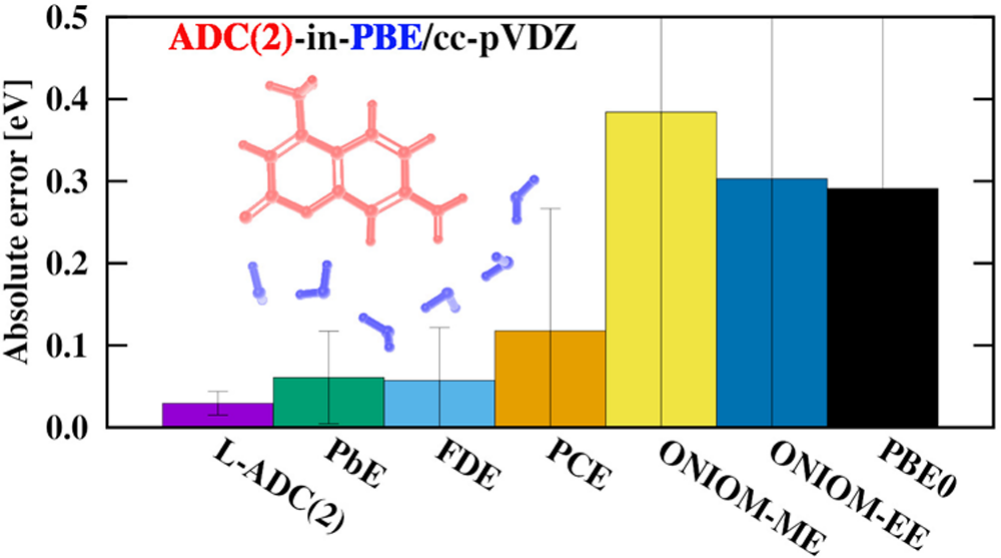

The performance of multilevel quantum chemical approaches, which utilize an atom-based system partitioning scheme to model various electronic excited states, is studied. The considered techniques include the mechanical-embedding (ME) of “our own N-layered integrated molecular orbital and molecular mechanics” (ONIOM) method, the point charge embedding (PCE), the electronic-embedding (EE) of ONIOM, the frozen density-embedding (FDE), the projector-based embedding (PbE), and our local domain-based correlation method. For the investigated multilevel approaches, the second-order algebraic-diagrammatic construction [ADC(2)] approach was utilized as the high-level method, which was embedded in either Hartree–Fock or a density functional environment. The XH-27 test set of Zech et al. [J. Chem. Theory Comput., 2018, 14, 40282990611110.1021/acs.jctc.8b00201] was used for the assessment, where organic dyes interact with several solvent molecules. With the selection of the chromophores as active subsystems, we conclude that the most reliable approach is local domain-based ADC(2) [L-ADC(2)], and the least robust schemes are ONIOM-ME and ONIOM-EE. The PbE, FDE, and PCE techniques often approach the accuracy of the L-ADC(2) scheme, but their precision is far behind. The results suggest that a more conservative subsystem selection algorithm or the inclusion of subsystem charge-transfers is required for the atom-based cost-efficient methods to produce high-accuracy excitation energies.

## 1 Introduction

Electronic structure modeling is becoming more and more important in the fields of chemistry, biology, and materials science as accurate predictions are more and more affordable for quite large systems. The computational costs of the traditional, highly accurate methods increase rapidly with the size of the system: the calculation time of the equation-of-motion^1^ or linear-response^2^ coupled-cluster method with single and double excitations (CCSD), which is commonly considered as a sufficiently accurate technique for most applications, scales as the sixth power with the system size. The expenses of CCSD quickly become intractable for most of chemists’ interest; thus, today’s routinely used method is the time-dependent density functional theory (TD-DFT)^3,4^ based upon the Kohn–Sham (KS) formalism,^5^ as its computational costs scale as the fourth power of the system size if a hybrid functional is employed. On the other hand, the accuracy of TD-DFT is rather limited^6,7^ in the case of Rydberg states or excitations with charge transfer character. A popular choice to overcome the failures of TD-DFT is the systematically improvable algebraic diagrammatic construction (ADC) technique,^8^ but its second-order variant [ADC(2)], which has the lowest computational cost, still scales as the fifth power of system size. As a result, it can only be used for medium-sized molecules, for example, typical organic dyes.

However, it is essential to model the excited states of extended systems, for instance, DNA-related excited-state processes and DNA–chromophore interactions.^9−13^ To circumvent the scaling of traditional methods, one can partition a large system at the atomic level into a chemically relevant (active) subsystem, which is treated with a high-level, costly method, and an environmental subsystem, where a fast, more approximate method is employed. The well-known quantum mechanics/molecular mechanics (QM/MM)^14,15^ technique utilizes this approach, but “our own N-layered integrated molecular orbital and molecular mechanics” (ONIOM)^16^ scheme also has these basics. In these models, the borders of the subsystems are usually handled by capping (link) atoms to saturate dangling bonds, and the environmental subsystem can polarize the active subsystem through point charges. They are efficient and simple to implement as there is no need to modify the quantum chemical code, and the subcalculations can be easily parallelized. Their downside is that the subsystems can only be separated effectively along single, apolar bonds by link atoms, the point charges can overpolarize the electron density of the active subsystem if they are spatially close, and it is not always straightforward to select the atoms of the active subsystem.^17−19^ Note that these effects can be more pronounced for excited-state calculations. Moreover, the predefined or generated point charges, which are usually parametrized to reproduce the ground-state electron density, may not be the most suitable representation of the environment.^20^ In the case of the ONIOM framework, the computation of high-energy excitations, which are necessary for full spectrum simulations, also becomes problematic since the order of states of various subsystems may interchange, and the simple subtraction technique may use inappropriate states for the extrapolation.^21,22^

One possible solution to the problems caused by point charges and link atoms is the projector-based embedding (PbE) technique of Manby and Miller,^23^ which allows for the combination of high-level DFT or wave function theory (WFT) methods with cost-effective DFT methods. The algorithm, which has also been greatly improved in the past decade,^24−32^ follows a top-down strategy: after solving the low-level KS equations of the whole system, the KS molecular orbitals (MOs) are localized, and the system is split up at the MO level. Subsequently, a high-level calculation is performed in the constant potential of the environment, while the MOs of the environmental subsystem are kept fixed through projection. In the case of excited states, the problem of the virtual subspace localization may arise, but the method has been successfully applied with the fragment localization scheme of Mayhall and Claudino^33^ by Jagau and Parravicini.^34^

Another multilevel approach is the frozen density embedding (FDE) theory of Weselowski and Warshel,^35,36^ which follows a bottom-up strategy: the system is partitioned into many subsystems—typically each solvent molecule around a solute is considered as a separate subsystem or fragment—and then the electron density of the predetermined fragments of the environment is calculated independently. Finally, the potential of the environment at the active subsystem is constructed as the sum of the fragment potentials, and an active subsystem calculation is carried out in the presence of the embedding potential. This approach is less demanding compared to the PbE technique since no calculation is required on the entire system. On the other hand, sufficient accuracy can only be ensured for noncovalent fragments because the nonadditive kinetic energy potential, which is an artifact of the overlapping environmental MOs, is not known. Note that previous studies showed^28,37^ that it is necessary to use a sizable active subsystem to minimize the error of the QM/MM and PbE calculations, and as a result, the overall expenses of such schemes are typically dictated by the costs of the high-level calculation.

Despite the improved characteristics of PbE and FDE over the QM/MM and ONIOM approaches, the manual selection of atoms of the active subsystem is still not avoided, and the results may largely depend on chemical intuition. In contrast, the subsystem formation of today’s most advanced local correlation techniques^38−46^ is solely based on the localization of MOs and the inclusion of important orbitals is fully automatic; thus, the most costly calculations are only performed in the most important but reduced subspaces. The errors of the local correlation schemes are, as compared with the canonical methods, marginal if an appropriate set of subspace truncation parameters is applied, while the computational costs are reduced by at least an order of magnitude. However, these algorithms can also be classified into the top-down class of layering approaches, because their first step is to solve the Hartree–Fock (HF) equations, which can have substantial costs even if cost-reduction techniques are utilized.^47−49^

Overall, it is not clear which of these approaches has the best trade-off between accuracy and computational cost for noncovalent systems. Since in this case, the selection of the chemically relevant region is straightforward, the focused models may become competitive with local correlation approaches. Thus, in this study, we examine the performance of various ONIOM, PbE, FDE, and local correlation techniques for the calculation of excitation energies on the XH-27 test set proposed by Zech et al.,^50^ which consists of medium-sized organic dyes surrounded by solvent molecules. This also provides an opportunity to test the effect of virtual orbital localization on excitation energies for the first time in the case of PbE approaches for a wider range of systems. In the following section, the theoretical basics of the employed methods are discussed, which will be followed by the assessment of their performance in extensive benchmark calculations.

## 2 Methods

In this section, the basics of the ONIOM, PbE, and FDE theories are presented, and our local correlation scheme is briefly outlined. Before the discussion of their theories, a few notations are introduced which will be used throughout the article.

The active and the environmental subsystems will be denoted with large capital letters A and B, respectively, and these will appear in the upper right index of the subsystem-specific quantities. Roman numbers I and II refer to the high- and low-level methods, respectively, and these will appear in the lower right index of a given quantity. The serial numbers of the corresponding electronic states 0, 1, 2, ..., *r* are displayed after the roman number, separated by a comma. The MOs (ϕ) are linear combinations of the atomic orbitals (AOs, χ). The bar symbol on top of the various quantities will denote a subsystem-restriction on the utilized AOs, furthermore, the usual ϕ_*i*_, ϕ_*j*_, ϕ_*k*_ and ϕ_*a*_, ϕ_*b*_, ϕ_*c*_ notations are reserved for the occupied and virtual MOs, respectively. For example, the ground-state density matrix of the active subsystem which is determined by a high-level method is denoted by , and this also means that the subsystem MOs are expanded in the AOs of the full (super) system. In the case of , the MOs are expanded only in the AOs that are positioned on the atoms of subsystem A. **R** will label the coordinates of atoms, while the coordinates of electrons will be denoted by **r**. Only closed shell systems will be discussed, moreover, atomic units and Dirac’s bra-ket notion will be used throughout.

### 2.1 ONIOM Scheme

In the original, simplest version of the ONIOM approach, which is a mechanical embedding (ME) type approach, the energy (*E*) of the system in its *r*th electronic state can be calculated by the following subtractive formula
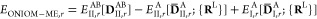
1where **R**^L^ denotes the coordinates of the link atoms (if any) and hydrogen atoms are typically used as capping atoms between carbon–carbon bonds. For the sake of simplicity, this dependency will be omitted, as link atoms are not used in this study. An important characteristic of the above formula is that the interaction energy of the subsystems is only accounted for at the low-level, and the density matrices of the active subsystem are calculated *in vacuo*. The excitation energy, ω_*r*_, of the ONIOM scheme can be obtained by subtracting the ground state energy from the energy of electronic state *r*:
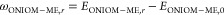
2

Note that utilizing the subtraction scheme for the excitation energies of the independent calculations gives the same expression,
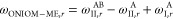
3where dependency of the excitation energies on the density matrix is omitted from the notation and similar formulas can be derived for the oscillator strengths. The ONIOM-ME procedure accounts for excitations in the whole system, including states with charge transfers between subsystems, and if the ω_II,*r*_ terms are omitted, we arrive at the simple vacuum embedding technique. A major disadvantage of this simple subtraction scheme is that it can be applied in a black-box manner only to the simulation of full spectra because eq 3 implies that the *r*th electronic state of subsystems A and AB are the same, but this is not guaranteed, especially for high-energy excitations. In this article, we will not deal with this problem as only the energetically lowest transitions were considered for such methods, and we recommend the work of Caricato et al. for the interested readers.^21,22^

A more advanced approach is the electronic embedding (EE) variant of ONIOM, where the subsystem energies have additional dependency:
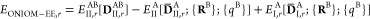
4Here {*q*^B^} represents the point charges that are associated with the atoms of subsystem B. These charges affect the electron densities of the active subsystem at both low and high levels through an embedding potential, ,
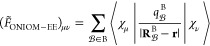
5where the summation runs over the atoms of subsystem B, and this potential is added to the core-Hamiltonian of the self-consistent field (SCF) equations of the active subsystem. Note that eq 4 is only valid when predefined point charges are employed, but the charges can also be generated on-the-fly using the ground-state density matrix of the full system calculation. The point charge representation of the environment usually has moderate effect on the outcome of the calculations, and it is not clear which atomic charge-determination approach performs the best.^20,51^ As before, the subtraction scheme can be used to obtain the excitation energies of the ONIOM-EE method from the subsystem excitations as
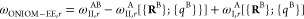
6where again, the density-matrix dependency of the excitation energies is omitted from the notation. In ONIOM-EE, similar to the ME version, all excitations of the system are described at least at the low level, including the charge transfer ones. However, the incomplete spectrum simulations could still be error-prone due to the possible inconsistencies in order of electronic states. The ONIOM-EE scheme can also be easily modified into a QM/MM-like model by omitting  and  at least for cases where the active subsystem excitations are the only relevant processes. This variant will be referred to as point charge embedding (PCE).

### 2.2 Projector-Based Embedding

Using the previously discussed system-partitioning approach, the ground-state energy of the full system using the PbE approach can be written as
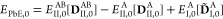
7where  and  are the self-consistent densities of the supersystem and the active subsystem, respectively, and  is the density of subsystem A computed from the self-consistent MOs evaluated for the supersystem as explained below. Note that all the density matrices are expanded over the AO basis set of the full system. In order to evaluate the above expression, the first step is to solve the KS equations of the full system using a low-level DFT technique:
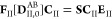
8Here **F**_II_ is the KS matrix of the low-level method, **C**_II_ is the MO coefficient matrix, **S** is the AO overlap matrix, **E**_II_ is the diagonal matrix which holds the eigenvalues of the operator, and  is the density matrix of the full system,
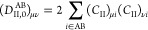
9calculated using the occupied MOs of the supersystem. The KS operator is iteratively constructed and diagonalized until self-consistency is reached, which is followed by the evaluation of . Subsequently, the occupied and virtual MOs are localized, and both the occupied and the virtual MOs are assigned to subsystem A or B. Using these localized KS MOs, **L**_II_, the density of the active subsystem can be determined as
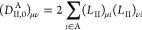
10which defines the corresponding energy term, . In addition, the low-level KS matrix for the active subsystem, , is also built in order to construct the embedding potential, , as
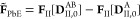
11

Using the following projector of the environmental subsystem
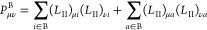
12the following equation is solved for the active subsystem
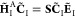
13where  and  contain the MO coefficients and the orbital energies of the reoptimized orbitals, respectively, and  is the modified Huzinaga-operator of the active subsystem. The latter is defined as
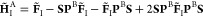
14and  is the embedded high-level operator, that is, the sum of the high-level operator of the active subsystem and the embedding potential:
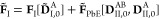
15Note that the tilde sign emphasizes that a quantity is calculated in the presence of an embedding potential.

After the MOs are reoptimized, the high-level energy of the active subsystem, , can be obtained, and a correlation calculation can be performed in the constant potential of the environment. Let us point out that we use the above operator because it keeps the sign of the eigenvalues of the environmental orbitals; thus, the orbital sorting is less troublesome during the second SCF run, where occupied and virtual orbitals are included in the projector.

Note also that the subsystem operator used also maintains the exact embedding feature of the Huzinaga operator,^27,52^ which means that the exact ground-state energy of the low-level method can be reproduced when the low-level technique is embedded into itself. The final ground-state energy of the PbE approach can be calculated as
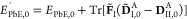
16where a first-order energy correction is added, which is derived from the Taylor-series of *E*_PbE,0_ expanded around the density of the active subsystem. After the ground-state energy is obtained, the transition energy of electronic state *r* can, in principle, be calculated as
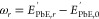
17but we do not attempt to calculate the first term on the right-hand side. Instead of trying to obtain system-wide transitions, we focus on the excitations of the active subsystem and evaluate the excitation energy as
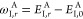
18

In practice, it means that an excitation energy calculation is carried out within the active subsystem in the presence of the embedding potential.

An alternative, less approximate version of PbE is also tested here. In that approach, the virtual orbitals are not localized, and the virtual MO space used for solving the equations for the active subsystem is the original virtual space of the supersystem. Then, the second term on the right side of eq 12 is zero, but the other equations also hold in this case.

### 2.3 Frozen Density Embedding

In the case of FDE, the only option is to calculate the quantities of the active subsystem, for example,  or , as the environment only appears as an embedding potential. On the other hand, it is more efficient because the whole system is split up into small, manageable fragments. Note that we will only give a very brief outline of the theory here, and the interested reader should see the work of Zech et al.^50^ and references therein.

The first step of FDE is to determine the electron density of each subsystem, and then the embedding potential is approximated as the sum of fragment potentials,
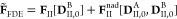
19where the last term is the potential derived from the intersubsystem-related, nonadditive energy terms, which can be defined generally as
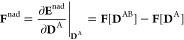
20

Specifically, the nonadditive potential can be split into four terms:
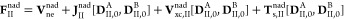
21where  is the potential of the nuclei of subsystem B,  and  are the nonadditive Coulomb and exchange-correlation (XC) potential, and  is the nonadditive kinetic energy potential. Note that the first two terms of the above equation are classical electrostatic terms and have an analytic form, while the last two ones are nonclassical and have to be approximated. The usual XC potentials of the DFT functionals can be used for , but an additional approximation has to be employed for , which is, in fact, an artifact of the overlapping electron densities of the monomers. When the fragments are not covalently bound, the choice of the functional form of  has a minor effect,; otherwise, the FDE model fails.

### 2.4 A Local Domain-Based Approximation

Our recently proposed local domain-based ADC(2) approach [L-ADC(2)]^46^ is also tested in this study. In this scheme, a compact, excitation-specific local domain is constructed for each transition, which includes all the important MOs for the excitation and the electron correlation. In addition, the virtual space of the resulting domains are further reduced, relying on the virtual natural orbital (VNO) approximation.^45^ This algorithm results in significant savings in computation time and enables us to extend the size of molecular systems that can be studied. Our numerical experience has shown that, using conservative predefined cutoff parameters, the mean absolute error introduced by this combined approach is only 0.015 eV. The speedups, of course, strongly depend on the nature of the excitation and electronic structure. Nevertheless, at least an order of magnitude savings can be expected in computation time. At the same time, 50-fold speedups were also gained even for systems of smaller than 100 atoms using triple-ζ basis sets with diffuse functions. Furthermore, as it was demonstrated, molecules of up to 400 atoms can be routinely treated. One of the major advantages of this scheme over the former ones is that it can be used in a black-box manner. On the other hand, the solution of the HF and configuration interaction singles (CIS) equations is required for the entire system. Accordingly, the CIS problem is the rate-determining step in this case. As the considerations regarding the approximations and their implementations are presented in refs (46 and 45) in detail, only a short overview of the approach is presented here.

The basic assumption of the local domain construction is that only a small subset of MOs contributes dominantly to a transition, and the number of these orbitals does not increase linearly with the size of the system. It is essential to keep in mind that the domain should contain all the MOs required for the adequate description of the ground and excited state simultaneously. To select the most important MOs involved in the excitation; first, the CIS eigenvalue problem is solved. Thereafter, the occupied MOs are localized, and projected atomic orbitals are constructed to span the virtual space. The CIS eigenvector is transformed to the basis sets obtained, and the importance of the corresponding orbitals is analyzed. This procedure selects the essential orbitals for the CIS wave function; however, it is well-known that the CIS solution could be an inappropriate description of the transition. Nevertheless, it can be assumed that the orbitals involved in the excitation are spatially close; for example, they can be found on the same chromophore group. Thus, if the domains are supplemented with the occupied and virtual MOs lying close to the selected orbitals, presumably all the important orbitals will be chosen. On the one hand, this step can improve the selection based on the CIS solution. On the other hand, as the excitations can also occur between two distant parts of the system, the occupied and virtual orbitals can be very far from each other. If only the orbitals selected relying on the CIS eigenvector were used in the calculations, the ground state correlation energy and amplitudes would be close to zero since important occupied and virtual MOs will be missing from the ground-state wave function. The aforementioned completion remedies this problem. During the local domain construction, the number of atoms, as well as the AO and auxiliary basis sets are also restricted. The resulting subspace is reorthogonalized and canonicalized. Accordingly, no changes are required in an existing ADC(2) code to calculate excited-state properties.

The virtual space of the resulting domain can be further compressed by invoking the VNO approximation. In this case, the virtual–virtual block of the one-particle density matrix is diagonalized. The eigenvectors of this matrix are the VNOs, while its eigenvalues are interpreted as the importance of the corresponding orbitals. Consequently, using a predefined truncation parameter denoted by ε_VNO_, the less important VNOs can be eliminated. Again, the VNOs should simultaneously be ideal for the ground and excited states. For this purpose, in our previous work, a so-called state-averaged density matrix has been introduced,^45^ which is obtained as the average of the approximate second-oder Møller–Plesset (MP2) and CIS with perturbative second-order correction [CIS(D)] density matrices. If the VNO approach is used without the local domain construction, 50% of the virtual orbitals can be safely neglected using triple-ζ basis sets with diffuse functions, while the average absolute error does not exceed 0.015 eV. The approximation is rather robust as the errors and the cost reductions are independent of the system size. Furthermore, it can be reliably used for all types of excitations. The local domain construction and the VNO approximation can be easily combined without any restriction. We note that the auxiliary basis can also be decreased using the natural auxiliary function approximation;^53^ however, it is out of scope in this study.

## 3 Details of the Calculations

### 3.1 Computational Details

The PbE and ONIOM approaches have been implemented in the Mrcc suite of quantum chemical programs and will be available in the next release of the package.^54^Mrcc was utilized in all other calculations as well. In this study, Dunning’s correlation consistent double-ζ basis set (cc-pVDZ)^55,56^ was used. In addition, the density-fitting approximation was applied to both the ground and the excited states, and the corresponding auxiliary basis sets of Weigend et al.^57−59^ were employed. The atoms of the organic dye were selected as the active subsystem in the case of the ONIOM, PCE, PbE, and FDE calculations. The PbE and ONIOM calculations utilized the Perdew, Burke, and Ernzerhof (PBE)^60^ functional and its hybrid version (PBE0)^61^ for the exchange-correlation functional as low-level methods. As the high-level method, the canonical ADC(2) model was employed in these calculations. The SPADE algorithm was used to localize the occupied and virtual subspaces in the case of the PbE calculations, and the relevant MOs were selected based on the change of the eigenvalues in the singular value decomposition procedure.^33^

The point charges were generated on-the-fly using the ground-state density matrix for the PCE technique and the electronic embedding variant of ONIOM. The intrinsic atomic orbitals (IAO) scheme^62^ was utilized to construct atomic charges; moreover, Mulliken and Löwdin atomic charges^63,64^ were also used on the test set but excluded from the discussion because the IAO charges seem superior (see Supporting Information). To guarantee the integer charges of all subsystems, the atomic charges located on the active subsystem atoms are summed up, the integer charge of the active subsystem is subtracted, and the remainder is distributed equally among the atoms of the environment. After this charge-correction, the atomic charges of the active subsystem are set to zero.

The results obtained with the FDE technique were extracted from the paper of Zech et al.,^50^ where the PBE and HF techniques were used to generate the densities of the fragments, and the generated densities were expanded on the whole system (not solely on the monomers). The LDA^65,66^ and GGA97^67−69^ schemes were employed to approximate the nonadditive kinetic energy potential, and the PBE^60^ method was utilized for the exchange-correlation potential.

In all excited-state calculations, the core orbitals were kept frozen. The assignment of excited states was performed via the analysis of natural transition orbitals.^70^ The visualization of orbitals was done using MOLDEN.^71,72^ The error of the excitation energies is defined as the canonical reference subtracted from the computed value. The statistical error measures presented in the figures are the mean absolute error (MAE), standard deviation (SD), and the maximum absolute error (MAX). All of the computed excitation energies are available in the Supporting Information.

### 3.2 Molecular Systems

The XH-27 benchmark set proposed by Zech et al.^50^ contains chromophores of chemical interest in a wide range of environments. These environments include several small molecules capable of being donors and/or acceptors in hydrogen bonds: water, ammonia, methanol, formamide, formic acid, methyl cyanide, methanethiol, and bromine monofluoride are used as neutral environments, while methanoate, trifluoric acetate, and the ammonium cation are employed to account for charged environments. While the choice of the environments is clearly focused on hydrogen bonding, the distance between the environmental molecules and the chromophores also covers a broad spectrum. As chromophores, both ionic (xanthinyle anion, pyridiniumyl benzimidazolide) and neutral (7-hydroxiquinoline, xanthine, aminopurine, diketopyrrolopyrrole, uracil, benzaldehyde, and coumarin 120) species are considered in the test set. The system configurations are labeled by *nx*, where *n* is a number representing the chromophore, whereas *x* is a letter used to differentiate among different environments for a given chromophore. Geometries were taken from ref (50), which took some of the geometries from ref (73). The interested reader is referred to ref (50) for a more complete description of the test set.

## 4 Results and Discussion

### 4.1 ONIOM and PCE Results

**Figure 1:**
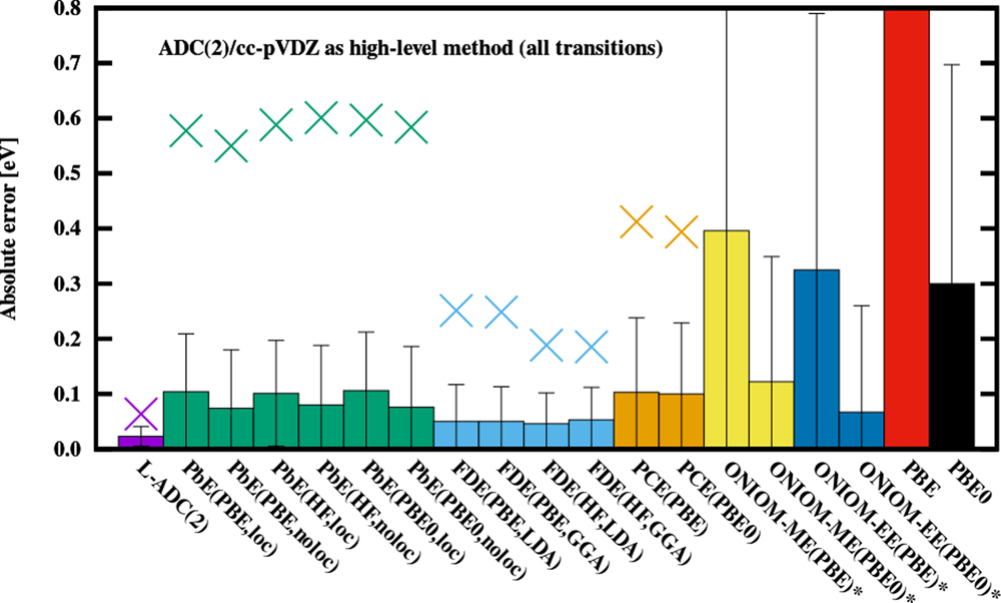
MAEs (bar heights), SDs (whiskers), and MAXs (crosses) of the discussed techniques, relative to canonical ADC(2)/cc-pVDZ reference excitation energies. For FDE, the first and second acronyms in the parentheses refer to the method used to generate the electron density and the functional which is utilized to approximate the nonadditive kinetic energy potential, respectively. For PbE, “loc” and “noloc” label the approaches where the virtual space is localized and not localized, respectively. The asterisk for the ONIOM calculations highlights that only the lowest-energy transitions are counted in the statistics.

**Figure 2:**
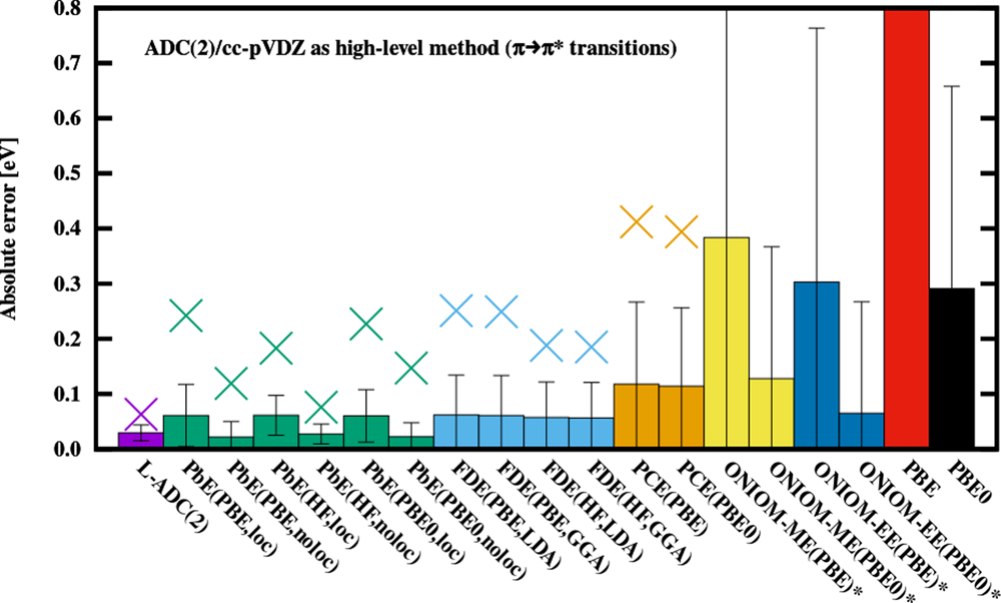
Error measures for the discussed techniques. Only π–π* transitions are used to calculate the statistics. See the caption of Figure 1 for further details.

**Figure 3:**
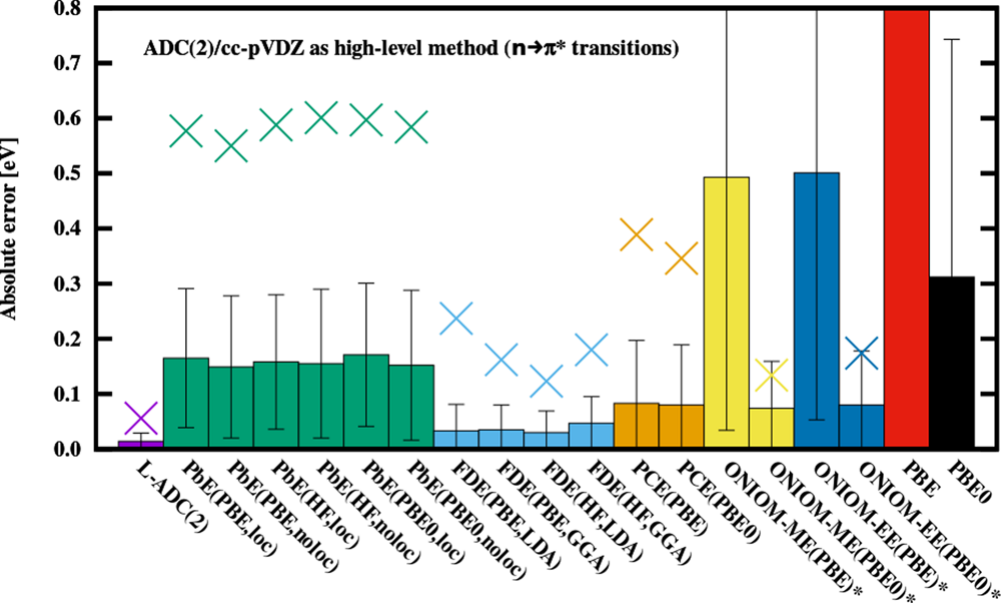
Error measures for the discussed techniques. Only *n*-π* transitions are used to calculate the statistics. See the caption of Figure 1 for further details.

The errors of the excitation energies, calculated with the various techniques, can be seen in Figures 1, 2, and 3. It is important to note that for the ONIOM approach, the figures only show the statistics of the errors for the lowest-energy transitions. As expected, the naive subtraction scheme may use different electronic states for the extrapolation, which produces unacceptable errors if higher excited states are also considered (over 0.2 eV on average, but the maximum error is over 2.0 eV). It is not shown, but this cannot be alleviated by choosing a better low-level method (in this case, PBE0 over PBE). However, for the lowest-energy transitions, the use of hybrid DFT greatly improves the results of both ONIOM schemes: the MAE drops from 0.40 (0.32) to 0.12 (0.07) eV for the ME (EE) technique; however, the SD is still around 0.2 eV and the maximum errors are larger than 1 eV. The EE approach looks somewhat more accurate compared to the ME technique, but this is not consistent for the different types of excitations. Also, considering the nature of the test systems, in particular, the presence of more or less strong hydrogen bonds, it is somewhat surprising that the ONIOM-EE method is shown to be competitive (at least, for the low-energy excitations) with the theoretically more advanced density-embedding schemes, although its robustness remains questionable. Still, these data indicate that the proper matching of electronic states with a more conservative active subsystem selection approach can produce a method better suited for routine applications.

Much more surprising is the finding that the PCE scheme, which is the simplest method, provides results comparable to the more advanced techniques, although it is of somewhat lower quality than the PbE or FDE approaches. (Note that all transitions, including high-energy excitations, are considered in the statistics.) Furthermore, the errors of PCE seem insensitive to the supersystem density matrix used, which means that this approach, using a PBE density for the determination of atomic charges, is the most cost-effective tool of all the investigated techniques.

### 4.2 PbE and FDE Results

Overall, the PbE scheme has a MAE of 0.10 eV, roughly twice of that of FDE. The same can be said about the SD and MAX values. One notable property of PbE is, as evident from the individual data presented in the Supporting Information, that the excitation energies are systematically overestimated. The choice of the method used in the supersystem calculation has no significant impact on the results, as even using HF instead of DFT leads to results of similar quality. The restriction of the virtual orbital space to the active domain introduces a small but definitely noticeable error of 0.02–0.03 eV. Investigating the outliers revealed that they are predominantly of *n* → π* character, therefore, a separate discussion of the π → π* and *n* → π* excitations will follow below supported by Figures 2 and 3.

The first observation is that the errors of the π → π* excitations are significantly smaller than those of *n* → π*. For the former, the MAEs are 0.02 eV for calculations using the complete supersystem virtual orbital space and 0.06 eV when the virtual orbitals are localized on the subsystems. This is comparable or even better than FDE’s MAE of 0.06 eV for these transitions, especially if one considers that the FDE results were obtained using the supersystem basis set. With this in mind, the PbE scheme offers similar accuracy at a lower computational cost with the localization of the virtual orbitals or increased accuracy at comparable cost when the complete virtual orbital space is used in the correlated calculation. While in absolute terms, the effect of localization is the same for the π → π* states as for the complete test set, in relative terms, the virtual orbitals of the nonactive subspace have significant contributions to the excitations with outlying error values.

For the *n* → π* transitions, the PbE technique is significantly less accurate than FDE, having up to five times larger average absolute errors. This can be attributed to the general nature of the systems included in the benchmark set as the central chromophore forms hydrogen bonds with the environmental molecules where the lone pair orbitals of the solute are inherently involved. While the localization of the virtual orbitals can have a sizable impact on a few selected excitations, it is statistically insignificant compared to the effect the frozen occupied orbitals of the environment can have on the error. A common property of the outliers is that in the reference full-system ADC(2) calculations, a considerable electron density is observed on the environmental molecules (CT character), while this is not possible in the PbE method.

### 4.3 Local Domain-Based Calculations

Finally, the overall performance of our L-ADC(2) approach^45^ is discussed. Again, the results are collected in Figure 1. Inspecting the errors obtained, the overall MAE is no more than 0.023 eV. In other words, the errors can be reduced by a factor of 2 compared to the FDE approximation, whereas the improvements are even more significant in comparison with the embedding approaches presented in this study. The precision of this approach is also outstanding: the lowest SD, precisely 0.018 eV, is achieved, while it is around 0.060 and 0.100 eV for the FDE and PbE schemes, respectively. Salient errors cannot be identified as the MAX is also favorable; the largest error is only 0.063 eV.

The errors are also fairly well-balanced regarding the excitation types as no significant differences in the accuracy can be observed between the π → π* and *n* → π* excitations (see Figures 2 and 3). The SDs and MAXs are practically identical for the two types of transitions, the former measure being 0.014 and 0.015 eV, while the MAX is 0.063 and 0.056 eV for the π → π* and *n* → π* excitations, respectively. Inspecting the MAEs, a somewhat larger difference can be observed with values of 0.030 and 0.014 eV for the π → π* and *n* → π* set, respectively. In other words, the error is twice as large for the former type of excitations. Nevertheless, with the difference being no more than 0.015 eV in absolute terms, this result is still acceptable. Interestingly, a similar tendency is seen for the FDE approximation as well, but this is more likely to be a result of a fortunate error cancellation of the FDE scheme as no correlation is observed between the excitation energies and the overlap of the densities.

The outstanding efficiency of L-ADC(2) relies on the significant savings in computation time achieved via local domain construction and the VNO approximation. The reduced subspace construction decreases the number of occupied and virtual MOs, while the resulting compact domain contains fewer atoms as well. Accordingly, the number of atomic orbitals and auxiliary functions is also reduced. On top of this, the virtual basis is further compressed by invoking the VNO approximation using ε_VNO_ = 3.5 × 10^–4^. The percentages of the retained orbitals and auxiliary functions are collected in Table 1.

**Table 1:** Percentage of Retained Occupied and Virtual Orbitals and Auxiliary Functions in the L-ADC(2) Calculations

	retained occupied MOs	retained virtual NOs	retained aux. functions
average	84.0	65.0	86.2
minimum	51.0	41.0	55.0
maximum	100.0	83.5	100.0

The discussion of the resulting basis set sizes is rather difficult due to the several factors that influence them. Nevertheless, about 15% of the occupied orbitals and auxiliary functions could be neglected. Due to the VNO approximation, the compression of the virtual space is more notable. In this case, 35% of the orbitals can be dropped. These favorable results are somewhat unexpected, considering that most systems contain fewer than 30 atoms. In the most adverse cases, the assembled domain contains all orbitals and functions, while the virtual subspace is only compressed moderately. For local excitations, on the other hand, half of the occupied MOs and auxiliary functions can safely be neglected, while the number of VNOs can be reduced by 60%. In these cases, 30–35-fold speedups can be realized in the ADC(2) part. These gains are presumed to be even more radical for larger systems.

The above findings clearly demonstrate the excellent performance of the L-ADC(2) approach. We would like to emphasize, however, that in this procedure the HF and CIS equations have to be solved for the entire supersystem. Accordingly, despite the significant cost reduction, such calculations could be more expensive for extended molecular systems than the embedding schemes. Nevertheless, our results indicate that the L-ADC(2) method can be an ideal candidate for benchmarking less reliable approximations for larger systems, where the canonical ADC(2) calculations are no longer feasible.

## 5 Conclusions

In this paper, various multilevel quantum chemical algorithms were investigated in order to test their performance in describing (dominantly local) excited electronic states. Since the computational costs of excited-state methods are generally higher than those of the ground-state approaches, the efficiency of the methods is of utmost importance when handling extended systems. This study covers a broad range of multilevel schemes, including the simple ONIOM-ME approach, the point charge (PCE, ONIOM-EE) and electron-density embedding (PbE, FDE) methods, as well as our local electron correlation-based technique. These approaches are applied to the systems of the XH-27 test set, where typical organic dyes interact with several solvent molecules forming different hydrogen bonds. The partitioning of such systems is trivial since the chromophore and the solvent molecules can be selected as active and environmental subsystems, respectively. As in the original paper of Zech et al.,^50^ the ADC(2) approach was used as the reference method for the assessment, and also the various approximate schemes used ADC(2) to describe the active subsystem.

In accordance with the expectations, our L-ADC(2) approach clearly proved to be the most reliable method, showing a significant improvement in efficiency compared to the canonical procedure despite the relatively small size of the test systems and the basis set. Although the accuracy of the system partitioning-based techniques was found to be close to L-ADC(2), their precision is far behind. Overall, the FDE, the PbE, and the PCE approaches proved to be more reliable, whereas the ONIOM schemes provided the least accurate data. Still, ONIOM is, in many cases, on par with the L-ADC(2) approach when only the lowest-energy transitions are considered, but the troubles associated with the matching of electronic states at different levels render its application rather difficult. It is also worth pointing out that, with the exception of ONIOM, every scheme provided better excitation energies than the TD-DFT method using either PBE or PBE0 functionals.

However, the assessment requires additional considerations because several technical details can have significant impact on the quality of the data. First, one has to be aware that the FDE results referenced here used the AO basis set of the supersystem; thus, one can expect that in a typical application where only the AOs of the active fragments are considered, the accuracy presumably would decrease. Second, the PbE scheme appears to be not very sensitive to restrictions of the virtual subspace, which could result in more efficient calculations as the number of virtual MOs usually outweighs that of the occupied ones. This and the fact that PbE can separate systems through covalent bonds present a serious advantage compared to FDE.

It can be asserted as a summary that more accurate results, as was shown for the ground-state calculations, require a more conservative system partitioning approach for the investigated methods because strong hydrogen bonds could form an inseparable part of the active subsystem. This also draws attention to the description of charge transfers between subsystems because chemical intuition could fail in predicting the most important MOs in specific cases. Thus, improved versions of the ONIOM-EE and the PbE approaches would present a more competitive alternative to the L-ADC(2) method. Based on the excellent observed accuracy of L-ADC(2) as well as the favorable cost requirements of PCE and PbE, we anticipate that the combination of these approaches in future studies can present efficient alternatives for modeling extended systems in their excited states.

## Supporting Information Available

Computed excitation energies (XLSX)
